# Neural Correlates of Switching Attentional Focus during Finger Movements: An fMRI Study

**DOI:** 10.3389/fpsyg.2012.00555

**Published:** 2012-12-14

**Authors:** Kristin M. Zimmermann, Matthias Bischoff, Britta Lorey, Rudolf Stark, Jörn Munzert, Karen Zentgraf

**Affiliations:** ^1^Institute of Sport and Exercise Sciences, University of MuensterMuenster, Germany; ^2^Bender Institute of Neuroimaging, Justus Liebig UniversityGiessen, Germany; ^3^Institute of Sport Science, Justus Liebig UniversityGiessen, Germany

**Keywords:** attentional focus switch, finger tapping task, fMRI, premotor cortex, intraparietal lobule

## Abstract

Research on motor-related attentional foci suggests that switching from an internal to an external focus of attention has advantageous effects on motor performance whereas switching from an external to an internal focus has disadvantageous effects. We used functional magnetic resonance imaging to investigate the neural correlates of switching the focus of attention. Two experimental groups were trained to apply one focus direction – internal or external – on a previously learned finger tapping sequence. Participants with an internal focus training were instructed to attend to their moving fingers; those with an external focus training were instructed to attend to the response buttons. In the first half of the experiment, participants performed with their trained focus, in the second half, they were unexpectedly asked to switch to the untrained attentional focus. Our data showed that the switch from a trained internal to an unfamiliar external focus of attention elicited increased activation of the left lateral premotor cortex (PMC). We propose that this activation can be linked to the role of the PMC in action planning – probably indicating a facilitation effect on selectional motor processes. Switching from a trained external to an unfamiliar internal focus of attention revealed enhanced activation of the left primary somatosensory cortex and intraparietal lobule. We interpret these modulations as a result of the amplifying influence of afferent information on motor processing when asked to attend internally in a motor task after being trained with an external focus.

## Introduction

In sports performance, athletes have to deal with a multitude of contextual demands while moving. For example, soccer players need to screen the pitch for teammates situated in several locations while concurrently shielding the ball from their opponents (Memmert, 2009). This warrants contingent changes in attentional focus direction to optimize motor performance. The influence of the focus of attention on movement has preoccupied research for over a decade, and there is accumulating evidence regarding the effects of discrete attentional foci on performance (e.g., Beilock et al., 2004; Gray, 2004; Ford et al., 2005). The most robust finding in this literature is that the instruction to concentrate on movement effects in the environment (external focus) enhances motor performance and learning. Transferred to the example of a soccer player, the player can either focus on his moving legs during shielding or he might focus his attention on the ball movements. Focusing attention on moving body parts (internal focus) seems to disturb motor processes because it commonly results in performance and learning decrements (see Wulf, 2007 for a review). When subjects in an experimental setting are asked to switch between an internal and an external focus of attention, the focus to be switched to seems to be the relevant one for performance (Wulf et al., 2001a; Ehrlenspiel et al., 2004; Weiss et al., 2008; Weiss, 2011). Thus, the instruction to switch from an external to an internal (EI) focus is more likely to elicit detrimental effects on task performance than the converse focus switch (IE).

Sticking to an external focus of attention over a longer period of time seems beneficial for movement production, but still sports experts switch their attentional focus on the field, e.g., triggered by a coach instruction. Recently, Bernier et al. (2011) examined the attentional focusing behavior of expert golfers in the field. Their qualitative analysis revealed that during the preparation and execution routine of a shot, experts applied a series of attentional foci, for example, with regard to content, to characteristics, or to whether the attended feature was real or imagined. Further research has identified a distinction between deliberate and stimulus-driven attention: switching between different attentional foci apparently seems to be an indispensable skill to adapt effectively to various kinds of environmental contexts and demands (Gopher, 1993; Memmert, 2009).

The findings described above strongly suggest that manifold factors influence attentional operations. This raises the question regarding which are the critical differences in motor processes when switching between an internal and external focus of attention. To address this question, research has started to elucidate the underlying mechanisms mediating the effects of these two attentional foci (e.g., Zentgraf and Munzert, 2009; Wulf and Lewthwaite, 2010). Using functional magnetic resonance imaging (fMRI), Zentgraf et al. (2009), for example, explored the brain structures involved in finger movements by comparing the two distinct attentional foci in a between-subject design. They observed an increase in blood oxygen level dependent (BOLD) response under an external focus of attention in the primary motor cortex, primary somatosensory cortex, and the insular region of the left hemisphere. They suggested that these findings reflected augmented sensory processing of tactile information when focusing externally on the buttons, which had to be pressed without visual control in the finger-moving task. Further, Zentgraf et al. (2009) propose more specifically that this augmented sensory processing had led to a modulation of integration mechanisms in neural sensorimotor systems. To our knowledge, however, the brain regions involved in switching from an internal attentional focus on movement to an external or vice versa have yet to be examined.

Whereas the left dorsal prefrontal cortex, left motor, and motor-association cortices, and bilateral parietal regions are considered to play a role in processing attention to action (e.g., Binkofski et al., 2002; Johansen-Berg and Matthews, 2002; Rowe et al., 2002), left-hemispheric posterior superior and anterior inferior parietal areas (i.e., supramarginal gyrus and regions ventral from the intraparietal sulcus) seem to be particularly relevant for attentional switches associated with a change in motor intention (Rushworth et al., 2003). A number of studies on the neural correlates of attentional switching have used visuo-spatial or auditory paradigms. In these experiments, shifting attention leads to activation of a frontoparietal network including the temporoparietal junction (Wager et al., 2004; Corbetta et al., 2008; Shulman et al., 2009). This network seems to be connected in turn to subcortical structures such as the basal ganglia, which are thought to modulate frontal connectivity to modality-specific association cortices (Robbins, 2007; van Schouwenburg et al., 2010). The main function of this attentional network is supposed to be the alteration of processing mechanisms that subserve the selection and optimal preparation of action along with the regulation of afferent input (Korsten et al., 2006). A switch in attentional focus direction would require the inhibition of processes associated with concurrent attentional operations in order to substitute them with computations of the novel response (Kübler et al., 2006). Altogether, an attentional focus switch is proposed to change neural processing on the task that precipitates in activational changes of the outlined brain regions.

The present study analyzes the second half of an experiment published by Zentgraf et al. (2009) and compares both parts. The objective of our study was to examine the neural structures involved in a switch of the attentional focus on an overlearned finger sequence. All subjects were trained in the application of one focus direction (internal or external) prior to the scanner session. The training resulted in two experimental groups, one familiar with the internal and the other familiar with the external focus of attention. The prelearned movement task consisted of a finger sequence with 16 key presses. After performing one-half of the trials under the trained focus in the scanner, participants were unexpectedly instructed to switch their attentional direction to the respective unfamiliar focus. Thereby the two groups differed in the kind of focus switching. The switching from a trained internal focus on the moving fingers to an untrained external focus on the response buttons (IE) occurred in one group, while switching from a trained external focus to an untrained internal focus of attention EI took place in the second group. In accordance with Zentgraf et al. (2009), we expected to find elevated BOLD response in brain regions associated with sensorimotor processing for the IE switch. Therefore, we specifically tested for statistically significant differences in BOLD response between the two focus conditions within both groups in motor areas, i.e., precentral gyrus (the primary motor cortex, premotor cortex (PMC), and supplementary motor area), as well as in the primary somatosensory, and the inferior parietal cortex (i.e., supramarginal gyrus and regions ventral from the intraparietal sulcus). Furthermore, for both groups, we expected higher activation in brain areas linked to attention switching processes when participants changed their attentional focus (Wager et al., 2004; Corbetta et al., 2008; Shulman et al., 2009). Therefore, we additionally tested for activation differences in those parts of the inferior, middle, and superior frontal region, which were directly adjacent to the precentral gyrus.

## Materials and Methods

### Subjects

Data of 31 participants was analyzed in our study. Participants were students from the University of Giessen (15 women, 16 men; age *M* = 24.7; SD = 2.9). Analysis of the first part of this experiment has been published by Zentgraf et al. (2009). All subjects were right-handed according to the Edinburgh Handedness Inventory (Oldfield, 1971) and screened to rule out any medication or illness that might influence cognitive or motor function. After data collection the 32nd subject was excluded from data analysis because of her experience in playing a musical instrument. All participants gave their informed consent and were reimbursed with 20 Euro or course credits. Experimental routines complied strictly with the Declaration of Helsinki.

### Training

On the day before the scanner session, participants completed a specific training procedure realized in Presentation software (Neurobehavioral Systems, Albany, CA, USA). The aim of the training was to habituate either an internal or an external focus of attention while performing a finger tapping sequence. This sequence comprised 16 key presses on a conventional response box with one specific key for each of three fingers (sequence order: ring–middle–index–ring–index–middle–index–ring–ring–middle–index–ring–index–middle–index–ring). All training was performed outside the MRI scanner. The training procedure started by presenting the entire sequence on a computer screen. Subsequently, participants conducted the sequence in parts. Initially, blocks of four key presses were presented and performed once. Following that, a block consisted of eight presses and participants again performed it once. Then, after twice performing the whole sequence of 16 presses in the correct order, an additional 30 correct trials had to be accomplished. If the first two inputs of the sequence (the whole 16 presses) were not entered in the correct order, participants had to start again with the training procedure from the beginning. In the last section of the training, participants were instructed to focus attention on either their moving fingers or the keys they had to hit. They were told to close their eyes prior to trial start and perform 50 trials correctly under their specific focus condition. Subjects thereby trained solely one of the two attentional foci and did not become acquainted with instructions for the other focus in the training session.

### Experimental design and procedure

#### Conditions

In the scanner session five conditions were implemented in two consecutive runs. Data of the first run has been analyzed in Zentgraf et al. (2009). The runs differed in the version of the focus condition, internal focus of attention (IN), or external focus of attention (EX). Both runs included a move-only (MO) task, a dual task (DT), and rest periods (REST). For the focus condition, participants were instructed to concentrate on either their fingers (IN) or on the keys of the response box (EX), while pressing the sequence learned in the training session. In MO, however, they were instructed to conduct the sequence without receiving any attentional instructions. DT was composed of performing the sequence while counting auditory signals (no attentional instruction was provided here). One to five uniform tones were presented via a high-quality stereo headphone set (compatible for use in the scanner). After DT, the number of tones heard had to be indicated on a visual analog scale after sequence execution. For this purpose, subjects used the same three fingers and keys of the response box as for sequence pressing. During a rest condition (REST), participants laid still in the scanner with their eyes closed until a sound signal was presented. Until the end of the first run, participants were familiarized with only one of the two attentional foci included in this experiment (internal or external). After the participants had completed the first run, the experimenter verbally explained the upcoming new attentional focus, and subjects were asked to confirm their comprehension. The verbal instruction given was standardized and paralleled to a further instruction presented visually at the beginning of the second run. In the experimental session inside the scanner, the order of the experimental conditions was randomized for each subject.

#### Trials

Directly prior to the scanner session (1 day after the training session), participants recalled the sequence and were familiarized with IN or EX, the other experimental conditions, and the REST condition outside the scanner. In the experimental session inside the scanner, the order of the experimental conditions was randomized for each subject. A trial consisted of the visually presented instruction followed by sequence execution under the specified condition. After the instructional text faded out, participants closed their eyes and performed the sequence according to the respective condition (during REST they did not move but likewise kept their eyes closed). Subsequently, after finishing the sequence, they opened their eyes and waited for the next instruction. At the end of DT, the rating was performed. Instructions and rating scales were presented by a mirror mounted on the head coil that depicted the display of a screen situated behind the subject’s head.

In each of both runs, participants performed 16 focus trials (IN or EX), 8 MO, 8 DT, and 8 REST trials. For each trial a block of 16 s was implemented in the stimulation protocol, the finger sequence was performed in about 8 s by participants on average. At the end of each trial, participants heard a tone instructing them to open their eyes. At the beginning of the first run, an anatomical (T1) volume and a field map were acquired. Then participants were habituated to the scanner noise and perception of the signal sounds was assured in a 4 min protocol. Duration of the experiment sequence itself was 14 min. In total the first run lasted 18 min plus 8 min for T1 data and a field map acquisition. All in all, the second run of the scanner session included 16 focus trials (IN or EX), 8 MO, 8 DT, and 8 REST trials with a total duration of 14 min.

### Behavioral data analysis

A logfile was recorded during the entire scanner session. This was used to test for possible differences in sequence duration and the number of sequence errors between the two runs for both focus groups. To make sure that participants pressed the sequence in a uniform rhythm, we also controlled for the time intervals between successive key presses. Statistical testing was performed with analyses of variance (ANOVAs) and *t*-tests for dependent measures.

### fMRI data

Data acquisition was carried out with a 1.5 T SIEMENS scanner (Symphony, Erlangen, Germany) using a standard head coil. For anatomical data, we obtained a T1-weighted whole-brain volume of 160 sagittal slices (slice thickness = 1 mm) using a three-dimensional gradient echo pulse sequence (MPRAGE). During the first and the second run of the scanner session, functional data were acquired as T2*-weighted echo planar images (EPI) with the following parameters: voxel size = 3 × 3 × 4.5 mm, 25 axial slices, TR = 2.5 ms, TE = 55 ms, 458 volumes in the first run, 347 volumes in the second run, flip angle = 90°, and matrix size = 64 × 64. Axial images were positioned parallel to the anterior-posterior commissure (AC-PC). Preprocessing and analysis of functional data was realized with SPM 8 (www.fil.ion.ucl.ac.uk/spm). Images were unwarped and corrected for head motion (realigned) in three translational and three rotational directions. For slice timing adjustment, the middle slice of each run was used as reference image. Spatial normalization to the Montreal Neurological Institute (MNI) template was carried out and images were smoothed with a 9 mm full width, half maximum (FWHM) isotropic Gaussian kernel. For each subject, a voxel-wise general linear model (GLM) was created.

Specified regressors were the following: task performance with focus (IN or EX), MO task, DT, resting phases (REST), and presentation of the instruction screens. Six further regressors specified head movements. A canonical hemodynamic response convolved with a delayed box-car function served for modeling the BOLD response. In a first-level analysis, for each run contrast images were calculated for focus (IN or EX) > REST (of the according run), focus > MO (of the according run), and focus > DT (of the according run). These contrast images were entered in a group (second-level) analysis assuming inter-individual random effects. For both groups, one group with IN in the first run, the other with EX in the first run, the second run was analyzed via one-sample *t*-tests to investigate global activation differences between conditions after the switch, i.e., for comparison of the versions of focus, not the switching itself (for analysis of the first run, see Zentgraf et al., 2009). Probabilities were corrected for multiple comparisons by a family wise error routine (FWE, *p* < 0.05) on the voxel level and labeled with the “SPM8 Anatomy Toolbox” procedure (Eickhoff et al., 2007).

To assess the specific effect of focus switching from trained to untrained in two second-level analyses (one per group), the first-level analysis contrasts of the focus conditions minus REST were contrasted between the first run and the second run. In the second-level analysis groups of first-level contrasts were tested with *t*-tests; the first-level contrasts directly represented the effects of interest. In other words, the two versions of switches were analyzed in the contrasts “IN > REST vs. EX > REST” and “EX > REST vs. IN > REST,” further referred to as IE and EI. Here, paired *t*-tests with run as repeated measurement factor were used. Small-volume correction was set up for previously defined regions (see Introduction and list at section end). For the purpose of Region-of-Interest (ROI) analyses, we created masks based on the anatomical segregation of the MNI brain (MNI; normalized single subject, high resolution T1 volume) using MARINA software (Walter et al., 2003). The tests within the search volumes were carried out with a small-volume correction on the voxel level (FWE, *p* < 0.05). Concerning the frontal cortex, we only included masks of those parts which were directly adjacent to the precentral gyrus, as we were interested in the spatial distribution of activation differences close to the motor areas. The search volumes (ROI-masks) included the following regions: precentral gyrus, postcentral gyrus, inferior parietal lobule (separated into angular gyrus, supramarginal gyrus, and the remaining dorsal part), inferior frontal gyrus (IFG; opercular part), middle frontal gyrus (excluding the orbital part), superior frontal gyrus (dorsolateral part).

## Results

### Behavioral data

#### Differences between groups

The logfiles were scanned for errors in sequence order or number of sequence presses. Number of incorrect trials was analyzed as dependent variable. These trials were excluded from further data analysis of mean sequence durations.

A 2 (Group) × 2 (Run) × 3 (Condition) mixed ANOVA for errors with repeated measures for Run and Condition revealed no significant differences between Groups, *F*(1, 29) = 1.17, *p* = 0.29, η^2^ = 0.04, no significant effect for Run, *F*(1, 29) = 2.22, *p* = 0.14, η^2^ = 0.07, no significant effect for the Run × Group interaction, *F*(1, 29) < 1, a significant trend for the Condition × Group effect, *F*(2, 58) = 3.04, *p* = 0.06, η^2^ = 0.10, and a significant Run × Condition × Group interaction, *F*(2, 58) = 5.20, *p* < 0.01, η^2^ = 0.15. Mean errors for groups and conditions will be described for each group separately.

A 2 (Group) × 2 (Run) × 3 (Condition) mixed ANOVA for mean sequence duration with repeated measures for Run and Condition revealed no significant differences between groups, *F*(1, 29) < 1, no significant effect for Run, *F*(1, 29) = 1.67, *p* = 0.21, η^2^ = 0.06, no significant effect for the Run × Group interaction, *F*(1, 29) = 2.29, *p* = 0.14, η^2^ = 0.07, no significant Condition × Group effect, *F*(2, 58) < 1, and no significant Run × Condition × Group interaction, *F*(2, 58) < 1. Mean sequence durations for groups and conditions will be described for each group separately.

#### Externally trained group

During the first run, mean sequence duration of the externally trained group was *M* = 7.65 s (SD = 1.30) for EX, *M* = 7.13 s (SD = 1.30) for MO, and *M* = 7.00 s (SD = 1.32) for DT. For the second run, mean sequence duration was *M* = 7.59 s (SD = 1.44) for IN, *M* = 6.74 s (SD = 0.93) for MO, and *M* = 6.80 s (SD = 0.98) in DT. To test for significant differences in sequence duration of the first and the second run within the externally trained group, we computed a2 (Run) × 3 (Condition) ANOVA with repeated measures. No main effect of Run, *F*(1, 14) = 2.57, *p* = 0.13, η^2^ = 0.16, but a significant main effect for Condition, *F*(2, 28) = 17.24, *p* < 0.001, η^2^ = 0.55, was identified. The Run × Condition interaction revealed no significant effect, *F*(2, 58) = 1.62, *p* = 0.22, η^2^ = 0.10. The main effect of Condition was due to longer sequence durations for the focus conditions compared with MO and DT.

In the first run, participants showed mean errors in EX with *M* = 0.60 (SD = 0.91), in MO with *M* = 0.53 (SD = 0.64), and in DT with *M* = 0.80 (SD = 1.15). In the second run, mean error was *M* = 0.87 (SD = 0.83) in IN, *M* = 0.27 (SD = 0.59) in MO, and *M* = 1.13 (SD = 1.51) in DT. A 2 (Run) × 3 (Condition) ANOVA with repeated measures revealed no main effect for Run, *F*(1, 14) < 1, no main effect for Condition, *F*(2, 28) = 2.21, *p* = 0.13, η^2^ = 0.14, and no significant Run × Condition interaction, *F*(2, 28) = 1.48, *p* = 0.25, η^2^ = 0.10.

#### Internally trained group

The mean sequence duration in the first run for the internally trained group was *M* = 7.74 s (SD = 0.99) for IN, *M* = 6.98 s (SD = 1.05) for MO, and *M* = 6.95 s (SD = 1.07) for DT. In the second run, mean sequence duration was *M* = 7.82 s (SD = 1.17) for EX, *M* = 6.95 s (SD = 1.19) for MO, and *M* = 6.95 s (SD = 1.16) for DT. A 2 (Run) × 3 (Condition) ANOVA with repeated measures for Run and Condition showed no significant difference between runs, *F*(1, 15) < 1, a significant effect for Condition, *F*(2, 30) = 20.34, *p* < 0.001, η^2^ = 0.58, and no significant Run × Condition interaction, *F*(2, 30) < 1. The main effect of Condition was again due to longer mean sequence duration in the focus conditions compared with MO and DT.

Mean number of errors in the first run was *M* = 0.88 (SD = 1.15) for IN, *M* = 0.75 (SD = 0.93) for MO, and *M* = 1.19 (SD = 1.11) for DT. In the second run mean error was *M* = 1.88 (SD = 2.33) for EX, *M* = 1.19 (SD = 1.56) for MO, and *M* = 0.50 (SD = 1.03) for DT. A 2 (Run) × 3 (Condition) ANOVA with repeated measures revealed no main effect of run, *F*(1, 15) = 1.55, *p* = 0.23, η^2^ = 0.09, a significant effect for Condition, *F*(2, 30) = 3.41, *p* < 0.05, η^2^ = 0.19, and a significant Run × Condition interaction, *F*(2, 30) = 6.29, *p* < 0.01, η^2^ = 0.30. *Post hoc* tests showed that the number of errors was larger in the focus condition of the second run (EX) than in the focus condition of the first run (IN), *t*(15) = 2.66, *p* < 0.05, and that it was larger in DT of the first run than in DT of the second run, *t*(15) = 2.20, *p* < 0.05.

### fMRI data

**Figure 1:**
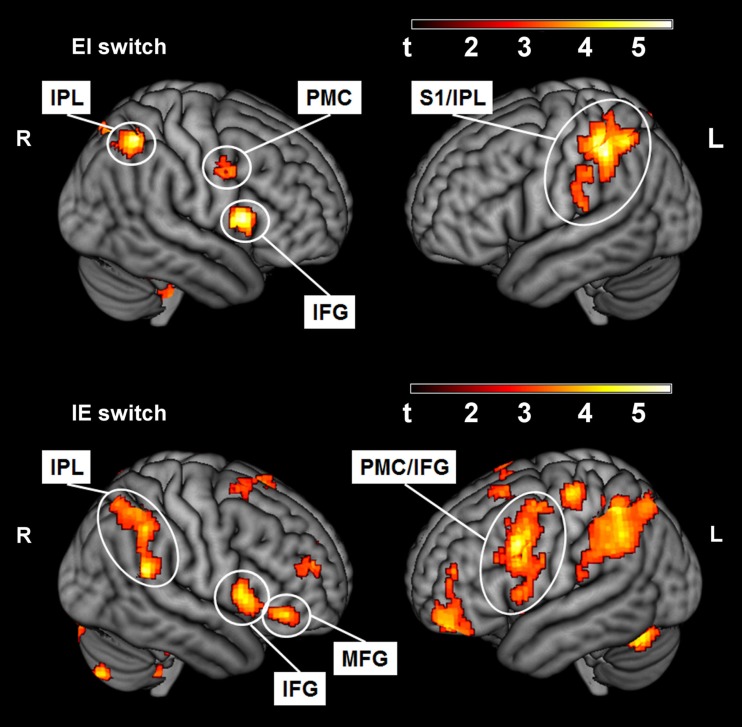
**Activated voxels (*p* < 0.01, *uncorrected*) for the within-group contrast of the EI switch (*IN* > *REST*, *second run* vs. *EX* > *REST*, *first run*; externally trained group) and the IE switch (*EX* > *REST*, *second run* vs. *IN* > *REST*, *first run*; internally trained group)**. IPL, Inferior parietal lobule (the dorsal part, excluding the supramarginal and angular gyri); PMC, premotor cortex (in precentral gyrus); S1, primary somatosensory cortex (postcentral gyrus); IFG, Inferior frontal gyrus, opercular part; MFG, Middle frontal gyrus (excluding the orbital part).

**Figure 2:**
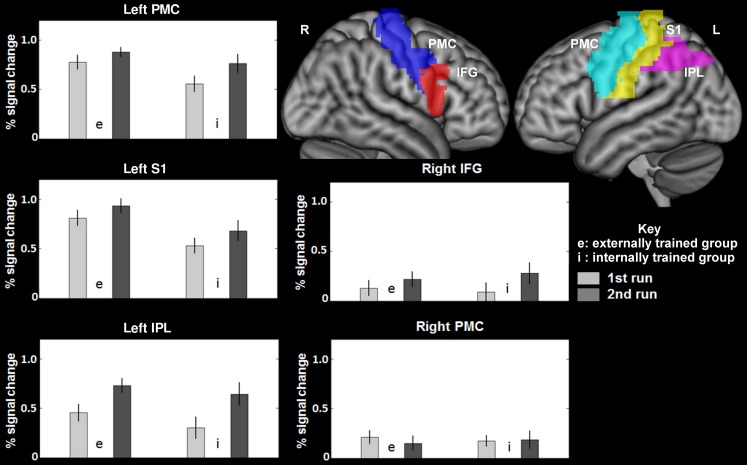
**Average percent signal changes within the search volumes of MARINA masks (Walter et al., 2003) for the left and right precentral cortex (premotor cortex), the left postcentral cortex (primary somatosensory cortex), the left intraparietal lobule (the dorsal part, excluding the supramarginal and angular gyri), and the right inferior frontal gyrus (opercular part)**. IPL, Inferior parietal lobule (the dorsal part, excluding the supramarginal and angular gyri); PMC, premotor cortex (in precentral gyrus); S1, primary somatosensory cortex (postcentral gyrus); IFG, Inferior frontal gyrus, opercular part.

To address the main question of the study, we compared the effect of switching IE and EI in both groups. Activation in the opercular part of the right IFG was found for both groups (see Table 1). The IE switch further elicited a greater BOLD response in the left IFG, the middle frontal gyrus, and in the left lateral PMC of the precentral gyrus (see Table 1, lower part). The EI switch showed specifically activation in the left primary somatosensory cortex (postcentral gyrus) and bilaterally in the dorsal part of the inferior parietal lobule (see Table 1, upper part). Detailed results of the ROI-analysis are presented in Table 1 and visualized in Figure 1. Figure 2 shows the percent signal changes for both groups and runs within selected search volumes. The percent signal changes were computed for the averages of the whole ROI, small volumes respectively, and are intended as descriptive additional information.

**Table 1:** **Overview of significant results of the ROI-analysis for the within-group comparisons of the two experimental groups (α < 0.05, *p*-values are *FWE-corrected*, cluster size threshold *k* > 5)**.

Cluster peak	H	*k*	MNI			*T*	*p*
			x	y	z	
**EI SWITCH**							
IFG, op.	r	71	63	17	16	5.93	0.006
Postcentral gyrus	l	252	−51	−37	55	5.93	0.015
IPL, dors.	l	448	−51	−40	49	5.87	0.011
IPL, dors.	r	133	39	−52	52	5.60	0.008
**IE SWITCH**							
IFG, op.	l	200	−57	14	31	4.65	0.020
IFG, op.	r	199	51	17	4	4.33	0.041
Precentral gyrus	l	581	−57	14	34	5.32	0.022

*For the externally trained group, we contrasted the internal focus condition of the second run with the external focus condition of the first run (*IN* > *REST, second run* vs. *EX* > *REST, first run*; *EI switch*). For the internally trained group, we contrasted the external focus condition of the second run with the internal focus condition of the first run (*EX* > *REST, second run* vs. *IN* > *REST, first run*; *IE switch*). IFG (op.), inferior frontal gyrus (opercular part); IPL, (dors.), inferior parietal lobule (the dorsal part, excluding the supramarginal and angular gyri)*.

**Figure 3:**
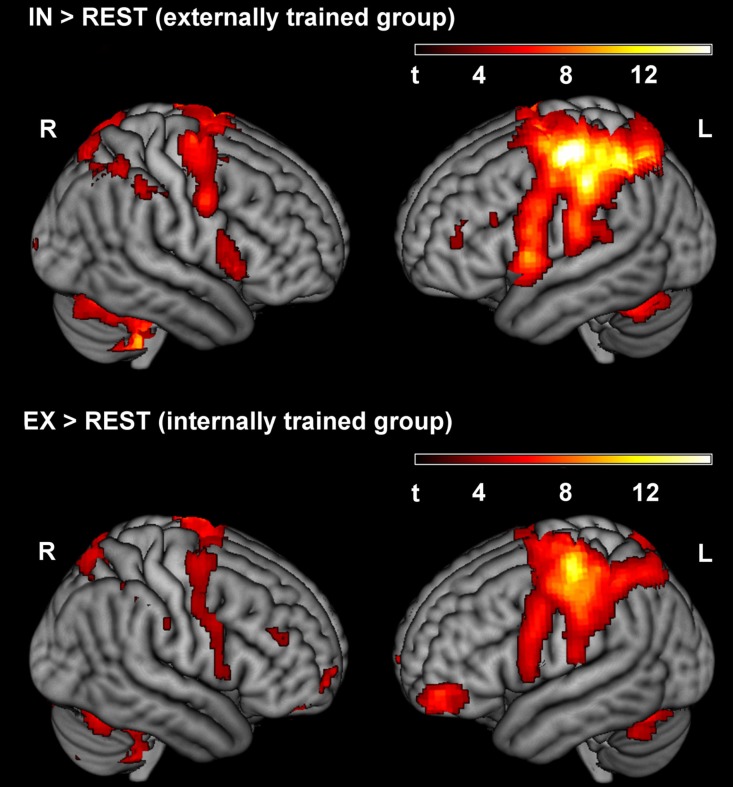
**Activated clusters for IN > REST and EX > REST of the second run, respectively (*p* < 0.001, *uncorrected*)**. During the second run, the externally trained group adopted the internal focus of attention, whereas the internally trained group focused externally.

In an analysis identical to the analysis of data of the first run published previously (Zentgraf et al., 2009), we computed separate contrasts of the focus conditions in the second run with the rest period (IN > REST and EX > REST). Figure 3 shows the results for the second run, outlining typical activation patterns in the left-hemispheric sensorimotor cortex and the right cerebellum when performing finger movements with the right hand. The ROI-analysis of the between-group comparison of the first run in Zentgraf et al. (2009) revealed higher activation for the externally trained group (contrast EX > REST vs. IN > REST) in the left primary somatosensory and motor cortex. For the opposite contrast (IN > REST vs. EX > REST), no significant activations were revealed.

Contrasting the focus condition and the MO condition of the second run, we found increased activation for the externally trained group (EI switch) across the frontal gyrus (see Table 2, upper part). For the internally trained group (IE switch) an effect was found in the superior frontal gyrus (see Table 2, lower part). The comparison of the focus condition of the second run with the DT condition revealed for the externally trained group (EI switch) higher activation in the left inferior parietal lobule (see Table 2, upper part). No effect was found for the internally trained group.

**Table 2:** **Overview of significant results of the ROI-analysis for the within-group comparisons of the two experimental groups (α < 0.05, *p*-values are *FWE-corrected*, cluster size threshold *k* > 5)**.

Cluster peak	H	*k*	MNI			*T*	*p*
			x	y	z	
**EI SWITCH**							
*IN* > *MO* vs. *EX* > *MO*							
IFG, op.	l	7	−48	11	1	4.79	0.027
MFG	l	296	−27	56	31	4.33	0.041
SFG, dors.	l	103	−24	56	34	5.93	0.019
*IN* > *DT* vs. *EX* > *DT*							
IPL, dors.	l	28	−30	−79	46	4.85	0.049
**IE SWITCH**							
*EX* > *MO* vs. *IN* > *MO*							
SFG, dors.	l	410	−18	56	19	7.35	0.001
*EX* > *DT* vs. *IN* > *DT*							
–							

*For the externally trained group, we contrasted the internal focus condition of the second run with the external focus condition of the first run after subtracting MO (*IN* > *MO, second run* vs. *EX* > *MO*, *first run*) or DT, respectively (*IN* > *DT*, *second run* vs. *EX* > *DT*, *first run*). For the internally trained group, we contrasted the external focus condition of the second run with the internal focus condition of the first run after subtracting MO (*EX* > *MO*, *second run* vs. *IN* > *MO*, *first run*) or DT, respectively (*EX* > *DT*, *second run* vs. *IN* > *DT*, *first run*). IFG (op.), inferior frontal gyrus (opercular part); MFG, middle frontal gyrus (excluding the orbital part), IPL (dors.), inferior parietal lobule (the dorsal part, excluding the supramarginal and angular gyri); SFG (dors.), superior frontal gyrus (dorsolateral part)*.

## Discussion

This study investigated the BOLD responses to a switch in attentional focus direction during the performance of a previously learned finger sequence. Our goal was to elucidate which brain regions would be involved in the switch – without prior notice – from a well-familiarized focus of attention to an unfamiliar one when performing the same finger movement task. To ensure the novel character of the unfamiliar attentional focus, we observed neural correlates associated with switching in two within-group analyses of separate groups. One group switched from an internal to an external focus of attention, the other group switched from an external to an internal focus of attention. We did not statistically test for differences between groups or switching directions due to possible serial-order effects. To our knowledge, whether the focus was switched equally successful from both types to the respective alternative in the two experimental groups could not be tested with our design. The possible asymmetry of switching severely restricts the explanatory power of between-groups tests, i.e., the switch on instruction may not be fully executed in one switch version. The specific value of this experiment is the controlled training of one focus and the level of novelty of the focus switched to. This should allow us to compare the two versions of switching, IE and EI. Our interpretations are only derived from the separate group analyses.

One fundamental problem when investigating effects of different attentional foci resides in the lack of a manipulation check to assess the applied attentional direction. Because focusing attention denotes a covert mental process, we did not control whether participants complied with the attentional instructions, but used verbal and visual cues at regular intervals to point out the current task and attentional focus direction to them.

Behavioral data showed that both groups performed effectively under their specific practice regime, which included a change of attentional focus. We did not find any significant effects for Group, Run, and the Group × Run interaction neither for number of errors nor for mean sequence duration. We therefore conclude that differences in neural activation rely on the specific attentional demands and are not a result of different performance outcomes within the groups and conditions.

### Attentional switching network

For both groups, we found increased activation in frontal and parietal areas when switching the attentional focus (EI and IE switch, see Table 1). These brain regions are part of a proposed supramodal frontoparietal attentional network (e.g., Driver and Spence, 1998; Corbetta and Shulman, 2002) that shows specific activation when switching attention to a new location (Wager et al., 2004; Corbetta et al., 2008). The functionality of the switching process is seen in the inhibition of neural operations on the prevailing stimulus–response pattern in order to replace either the stimulus or the response, thereby establishing a new stimulus–response map. Especially the right cortical hemisphere seems to play a fundamental role in the context of cortical inhibition operations (Robbins, 2007; Coxon et al., 2008). For both groups, it was especially the right IFG that increased its activity after the switch (EI and IE, respectively). Alongside its widely accepted function of suppressing motor responses to a predefined stimulus, there is substantial evidence that the right IFG is also involved in other tasks such as cognitive set switching or interference suppression (Konishi et al., 1999; Bunge et al., 2002). Recently, Dodds et al. (2011) have emphasized the importance of the right IFG for integrating updated sensory information in intended action plans. With regard to our experiment, we suggest that the realization of the attentional switch from a trained to a novel focus direction required the inhibition of cortical representations associated with the trained attentional focus. Furthermore, a novel connection between the instructed focus and the sequence performance had to be processed. Therefore, the co-activation of the right IFG with the attentional switch is considered to delineate ongoing interference in cortical processing, especially in association with the trained and the newly introduced attentional focus highlighting different sensory consequences.

### The EI switch

The switch from a trained external to an unfamiliar internal focus (EI, see Table 1 and Figure 2 upper parts) elicited higher activation of a large cluster in the left IPL expanding into the S1. The S1 receives afferent somatosensory input from the periphery (Blatow et al., 2007; Eickhoff et al., 2008). During movement, this upcoming sensory information is integrated into computational motor processing to adjust performance according to the motor plan in interaction with environmental demands (Rizzolatti et al., 1997; Dijkerman and de Haan, 2007). Together with the S1, the IPL plays a major role in sensorimotor integration processes (Rushworth et al., 1997; Filimon et al., 2009). Thus, we conclude that the neural activation pattern elicited by the EI switch is the result of a modulation of sensorimotor processing mechanisms. However, how does the EI switch in our study relate to sensorimotor processing?

In our experimental design, participants performed the finger-pressing sequence with eyes closed. Although precluding the visual input was tantamount to an even greater aberration from naturalistic sports settings, this enabled us to minimize sensory sources with a modulatory potential on brain activity. Thus, due to the lack of visual input, the only dynamic sensory impression consisted of haptic afferences from moving fingers and pressing keys. Although the sensory input of the first and the second run did not change, we suggest that the attentional instructions in our study differ in highlighting divergent submodalities of the haptic sense. Whereas the external focus directed toward the keys would aim at the tactile input derived from finger-key contact, the internal focus instruction to concentrate on the moving fingers would place more emphasis on the proprioceptive information available while moving.

Cutaneous and kinesthetic information is assumed to interact in the generation of a tactile shape impression or position sensing, and there are neurons in S1 sensitive to both tactile and proprioceptive input (Voisin et al., 2002). Nevertheless, there is accumulating evidence for the particular importance of tactile information for motor operations. For example, Symmons et al. (2007, 2008) compared the potential of movement-associated tactile and kinesthetic information to catch attention in an automated fashion. Participants put more weight on the informational content derived from touch than on the kinesthetics derived from the movement of their hand. Tactile input obviously exerts a strong influence on the capture of attentional resources. After attention has been directed toward tactile stimulation, it becomes very hard to shift it toward a different focus (Spence and Gallace, 2007).

Concerning underlying motor processes, Master and Tremblay (2009) found an increase in corticomotor excitability when subjects executed finger movements, thereby sensing movement-related tactile input of a 2-D shape as opposed to moving over a plain surface. Furthermore, the excitability substantially decreased when the subjects’ attention was drawn away from the movement execution, although tactile input remained present, revealing an interaction effect between the tactile stimulus and attention. Paying attention to tactile input obviously alters the integrational influence of somatosensory stimuli on motor processing mechanisms (Rosenkranz and Rothwell, 2004). Additionally, for the optimal computation of sequential hand movements, however, the inclusion of motor-related tactile input seems to be crucial (e.g., Goebl and Palmer, 2008).

During the training phase and the first run of our experiment, participants in the EI group repeatedly performed the sequence in contingency with focusing on the keys. Hence, their processing of the attended tactile information might have been integrated into cortical representations of sequence execution (e.g., Schneider and Shiffrin, 1977; Cohen et al., 1990). For the EI switch, the internal focus instruction to concentrate on the finger movements implicitly prompts the performer to blank out the tactile impact of the finger-key contact. It seems arguable that the persistent tactile perception of the keys might hamper the adequate adoption of the internal focus of attention. The urgency to ignore the attention-drawing and facilitating tactile sensing in order to adequately follow the internal instruction and focus on the motor-derived proprioception could therefore lead to an augmentation of the sensory site of movement processing as indicated by activation of the S1 and IPL (Noppeney et al., 1999; Burton and Sinclair, 2000; Rabin and Gordon, 2005; Kavounoudias et al., 2008). In this sense, we talk about a mechanism gradually adapting to the new focus direction instead of a prompt accomplishment of the attentional focus switch.

### The IE switch

Alongside the contribution of frontal cortex areas assigned to attentional reorientation processes, switching from a trained internal to a newly introduced external focus (IE, see Table 1 and Figure 1 lower parts) elicited increased neuronal activity in the left lateral PMC. The PMC is connected anatomically to a range of motor and motor-association areas, including the principal executive motor domain, namely, the primary motor cortex. Moreover, PMC projects directly to interneurons of the spinal cord (Chouinard et al., 2003). It is involved in the execution of finger sequencing (Halsband et al., 1993; Hlustik et al., 2002). Mainly the dorsal PMC is believed to play an important role during the selection of action (Schluter et al., 1998; Rushworth et al., 2003; Schumacher et al., 2003). These selectional processes are thought to depend on predictions about action-induced sensory events including the integration of momentary afferent input (Schubotz and von Cramon, 2003; Bubic et al., 2010; Stadler et al., 2012). Thus, for the purpose of selecting the adequate motor commands, PMC processing mechanisms are believed to rely on the attended sensory stimulus and its spatial location. When the focused stimuli are relevant for behavior, the selection through the dPMC is surmised to proceed mostly in an automated, stimulus-driven fashion. Depending on the context, however, this automatized movement generation appears to be modulated by controlling processes via neural connections of frontal cortex areas with the PMC (Cieslik et al., 2011). Contextual factors that are proposed to enhance the influence of executive control operations on motor selection include those requiring attentional reorientation such as changes in stimulus dimensions or task. In this case, attention-related brain structures such as frontal areas or the temporoparietal junction would modulate PMC processing.

In the course of the instructed IE switch, participants were asked to reorient their attentional focus. On the assumption that the two attentional foci of this study emphasize different sensory modalities, participants switching IE would shift from concentrating on their motor-evoked kinesthetics to focusing on tactile contact with the keys. This, in turn, is presumably followed by a change in motor processing, as indicated by the activation of the left lateral PMC. Furthermore, the lateral PMC has been demonstrated to be crucial for the establishment of mappings between sensory stimuli and motor responses (Cieslik et al., 2010; Amiez et al., 2012). Regarding the results of the present experiment, we hypothesize that the activated frontal brain areas reflect an ongoing process of attentional switching. Their functioning would reside in the endogenously controlled modulation of left lateral PMC selectional processes. The latter could be further described as a representational binding between the key-induced tactile afferents and motor operations for an optimization of sequence performance. Regarding the functional relevance of the contralateral PMC for selectional and executional motor computations, the activation of this brain region when switching IE could indicate a facilitation effect on motor processing mechanisms (Cieslik et al., 2010). Nevertheless, it is important to note here that because participants were trained extensively in sequence performance, we did not investigate any effects in relation with motor learning. Assumptions on motor facilitation are based on neural activational changes and therefore are more speculative in nature.

### Theoretical perspectives and conclusion

Within the theoretical framework of the constrained action hypothesis, Wulf et al. (2001b) have proposed an explanation for the beneficial effects associated with the adoption of an external focus of attention. This derives from the assumption that attending to external movement effects permits the motor system to conduct its processes in an automated fashion, whereas focusing on moving body parts seems to interfere with these automatic computational processes.

Nonetheless, we still do not know why attending environmental features compared to a focus on bodily movement seems to be beneficial for motor performance. To take a further step toward clarifying the underlying mechanisms in line with the results of this study, we refer to the biased competition (BC) model of visual attention (Desimone and Duncan, 1995; see also Smith and Schenk, 2012). The authors of the BC claim that the attentional potential of a sensory stimulus depends on its physical salience: the more salient, the greater would be its neuronal representation in sensory and motor systems. Sensory stimuli compete for representational resources within the sensorimotor system. This competition is outlined as a process of integrating the diverse representations, resulting in a single representation with the most salient stimulus accounting for the largest proportion. Finally, attention is defined as the result of these integrated representations. In addition, the competition can be modulated by endogenous control operations, thereby extending the influence of less salient sensory stimuli.

With regard to the results of switching EI, and in accordance with the BC, we propose that the activation in left S1 and IPL may be the result of an ongoing competition for attentional resources between the salient tactile and the less salient, but voluntarily focused, kinesthetic sensory modality (Burton and Sinclair, 2000; Symmons et al., 2007, 2008). In the same vein, activation of the left lateral PMC in association with the IE switch supposedly could lead to a facilitation of selectional motor processing, because the sensory modality with the greater potential to capture attention and the intended focus direction would converge. This suggestion could be supported by the fact that the activation pattern of the PMC seems to depend on the sensory modality focused (Schubotz and von Cramon, 2003). In varying situations, some sensory modalities may have greater motor significance than others, leading to different weighting or inclusion in motor processing. Nevertheless, whether the most salient sensory modality within a certain context simultaneously outlines the one with the greatest motor significance is something that remains to be clarified (Schubotz and von Cramon, 2003). Finally, sensory afferents may also constitute a prerequisite with regard to endogenously generated processes of motor intention (Cole, 2004). Overall, afferent sensory information seems to play an important role for motor processing mechanisms when switching the attentional focus between moving fingers and keys.

Future studies should examine whether the BC framework could be extended beyond visual stimuli and to more complex movement forms. Furthermore, they should also clarify how switches between internal and external foci of attention relate to sensory processing mechanisms and what is the role of salience for the underlying mechanisms or performance measures.

To summarize, this manuscript presents a first study of a switch in attentional focus direction during a finger tapping sequence. The results delineate the involvement of sensorimotor brain areas in processing switches between an internal and an external focus of attention and are speculated to emphasize the importance of the sensory modalities accentuated under the attentional foci.

## Conflict of Interest Statement

The authors declare that the research was conducted in the absence of any commercial or financial relationships that could be construed as a potential conflict of interest.
